# Hibiscus derived nitrogen doped carbon nanochains for visual pH sensing and catalytic methyl orange degradation

**DOI:** 10.1038/s41598-026-59561-0

**Published:** 2026-07-30

**Authors:** Hebat-Allah S. Tohamy

**Affiliations:** Cellulose and Paper Department, National Research Centre, 33 El Bohouth Str., P.O. 12622, Dokki, Giza Egypt

**Keywords:** Anthocyanin, *Hibiscus sabdariffa*, Density of states analysis, Carbon nano-chains, Dye sensor, Water treatment, Water sensing, Chemistry, Environmental sciences, Materials science, Nanoscience and technology

## Abstract

The rapid expansion of synthetic azo dye pollution requires the development of multifunctional nanomaterials capable of simultaneous real-time monitoring and active chemical remediation. Herein, we report the one-pot, microwave-assisted hydrothermal synthesis of nitrogen-doped carbon nano-chains (N-CNCs) using *Hibiscus sabdariffa* botanical waste as a sustainable precursor. Transmission electron microscopy (TEM) confirmed the fabrication of an interconnected, one-dimensional (1D) beads-on-a-string morphology composed of monodisperse beads (2.08–2.96 nm). Selected area electron diffraction (SAED) verified a short-range turbostratic amorphous carbon framework. The synthesized N-CNCs function as a high-performance dual-mode environmental platform, serving as a high-contrast naked-eye pH sensor and an ultra-rapid catalyst for methyl orange (MO) degradation. Catalytic trials demonstrated a clean, systematic elimination of the chromophore within seconds (< 1 s) at environmental extremes, reaching degradation efficiencies of 79.30% (pH 3) and 74.67% (pH 12). Computational insights from Density Functional Theory (DFT) and Density of States (DOS) analysis decoded the underlying quantum logic, revealing a dramatic collapse of the frontier molecular orbital energy gap (E_g_) from 8.5416 eV in native N-CNCs to an ultra-reactive 0.8816 eV within the hybrid matrix. DOS spectra confirmed intense orbital crowding near the Fermi level, which drives instantaneous, non-radiative intramolecular electron transfer for irreversible azo-bond cleavage. This metal-free, circular-economy platform successfully bridges real-time optical tracking with high-capacity chemical remediation, offering a highly competitive blueprint for advanced wastewater treatment.

## Introduction

The rapid expansion of global industrialization has brought with it a quiet but devastating environmental crisis in the form of synthetic dye pollution, with azo dyes like Methyl Orange (MO) serving as a primary culprit^1,2^. These robust organic compounds are widely used in textile, printing, and paper manufacturing, yet they are notorious for their intense color and complex chemical structures that resist natural degradation^1,3,4^. When discharged into water bodies, even at relatively low concentrations, they create a persistent barrier that blocks sunlight, disrupts aquatic photosynthesis, and introduces toxic, potentially carcinogenic byproducts into the ecosystem^5–7^. However, a significant gap exists in how we approach this problem; current remediation strategies often focus solely on the physical or chemical removal of the dye, overlooking a critical operational reality. Achieving mere visual clarification of the water is only half the battle, as the chemical state of industrial effluent is highly dynamic. Most remediation processes whether via passive adsorption or active chromophore degradation are extremely sensitive to the acidity or alkalinity of the environment^8–10^. Without an integrated, smart system capable of monitoring pH levels in real-time, treatment becomes inefficient or unpredictable. Precise pH control is not just a laboratory requirement but a functional necessity for effective remediation, as it dictates the surface charge of catalysts and the molecular stability of the pollutants themselves^8^. Therefore, the next generation of environmental solutions must move beyond passive filtration toward multifunctional platforms that can simultaneously sense the chemical environment and actively eliminate the toxic burden. Despite the advancements in environmental nanotechnology, current remediation methods remain tethered to significant practical and ecological limitations. Many traditional approaches rely heavily on metallic nanoparticles, such as the silver-based systems (AgNPs) which are prized for their high catalytic activity^11^. However, these metal-dependent strategies often face the stability-toxicity trade-off; they frequently require complex synthetic stabilizers to prevent aggregation and are prone to metal leaching, which can introduce secondary pollutants into the very water systems they are intended to clean^12–14^. Furthermore, the high cost of noble metals and the energy-intensive nature of their synthesis pose a major barrier to large-scale industrial application^15^. This has catalyzed a paradigm shift toward the principles of green chemistry, seeking out metal-free, bio-based alternatives that align with a circular economy. There is a growing demand for sustainable catalysts that can be derived from abundant natural precursors, offering a cost-effective and earth-friendly route to water purification. By moving away from resource-heavy metallic systems and toward carbon-based nanostructures, we can engineer materials that are not only safer for the environment but also inherently more stable and easier to recover.

In the search for more sustainable alternatives, Carbon Dots (CDs) have emerged as a superior class of nanomaterials, offering a unique blend of low toxicity, high biocompatibility, and exceptional optical properties that traditional metallic systems often lack^16–19^. Unlike their metal-based counterparts, these carbon-based fluorophores are inherently stable and easily synthesized from abundant organic sources, making them ideal candidates for large-scale environmental monitoring^20,21^. However, the true secret ingredient to elevating their performance from simple passive markers to active chemical agents lies in the strategic introduction of heteroatoms, specifically through nitrogen doping (N-doping)^22,23^. By integrating nitrogen into the carbon lattice, the electronic density of the material is fundamentally reshaped, creating a wealth of chemically active sites that serve as a bridge for electron transfer^24,25^. This electronic reorganization is what ultimately unlocks the latent catalytic power of the carbon framework, enabling the efficient degradation of resilient pollutants like methyl orange (MO)^26–30^. Furthermore, the evolution of these materials from conventional zero-dimensional (0D) isolated dots into one-dimensional (1D) nano-chains represents a significant morphological breakthrough. This beads-on-a-string architecture does not merely increase the available surface area for dye interaction; it creates a continuous molecular highway that facilitates rapid electron transport. By transitioning to this interconnected nano-chain morphology, the material transitions from a simple additive to a high-performance, multifunctional platform capable of driving complex chemical reactions with remarkable speed and precision. The selection of *Hibiscus sabdariffa* as a biological precursor is far from arbitrary; it represents a strategic alignment between nature’s molecular complexity and modern nanotechnology. Hibiscus is naturally endowed with a rich profile of organic nitrogen and anthocyanins, the very pigments responsible for the plant’s vibrant colors and its inherent sensitivity to environmental acidity^31,32^. By utilizing this botanical source, we tap into a pre-existing chemical library where nitrogen is already embedded in the organic matrix, simplifying the path to high-performance N-doped nanostructures. This natural logic allows the resulting materials to inherit the plant’s intrinsic pH-responsiveness, which we have effectively upgraded from a transient biological trait into a robust, stable, and highly sensitive nanomaterial capable of surviving harsh industrial conditions. Beyond the technical advantages, this approach champions the principles of a circular economy by transforming common botanical waste into a high-value smart tool. This transition from raw agricultural byproduct to a sophisticated multifunctional platform not only reduces the carbon footprint of nanoparticle synthesis but also proves that the most elegant solutions to modern pollution may well be hidden within the natural world’s own discarded materials.

While the utilization of agricultural waste for the fabrication of zero-dimensional (0D) carbon dots has been widely reported, the field remains constrained by the challenges of random aggregation, charge-carrier trapping, and an over-reliance on purely empirical optimization. The true novelty of this investigation lies in breaking past these boundaries through a coordinated architectural and quantum-theoretical approach. By moving away from isolated quantum structures, we report the directionally guided synthesis of interconnected, one-dimensional (1D) carbon nano-chains. This continuous beads-on-a-string framework establishes a high-conductance electronic highway that bypasses traditional charge traps. Crucially, rather than treating the resulting biomass matrix as a black box, we utilize Density Functional Theory (DFT) and Density of States (DOS) analysis to map the exact quantum logic driving the platform’s functionality. This reveals a profound collapse of the frontier molecular orbital energy gap (E_g_) and intense orbital crowding near the Fermi level upon interaction with the target chromophore. By combining this continuous morphology with deep quantum mechanical insights, this work moves beyond traditional passive biomass recycling to deliver a predictable, highly responsive, metal-free environmental tool that integrates real-time optical tracking with active chemical remediation^33^. In this present work, we report a significant leap in functional nanomaterials through the targeted synthesis of Hibiscus-derived nitrogen-doped carbon nano-chains, engineered to serve as a sophisticated, dual-mode environmental platform. Our innovation lies in the strategic transition from isolated quantum dots to an interconnected, one-dimensional nano-chain architecture, which acts as both a high-sensitivity optical probe and a robust catalytic agent. The smart hallmark of this material is its remarkable ability to provide high-intensity, naked-eye pH sensing; even when dealing with significant dye concentration of 10 mg/L, the nano-chains amplify the chromatic transition, making the chemical state of the water visible without the need for complex instrumentation. This visual clarity is seamlessly integrated with an active remediation phase, where the nitrogen-rich surface of the nano-chains drives the efficient catalytic degradation of MO. Crucially, the primary focus of this investigation is anchored on the high-intensity chromofluorogenic sensing mechanism, optimizing the N-CNCs as a smart visual indicator first, while exploring the steady-state degradation efficiency as a sequential environmental remediation step. This dual functionality simultaneously signaling the pH environment while systematically breaking down the pollutant has been rigorously verified through UV–Vis spectroscopy, confirming the rapid degradation of the azo bond. By merging real-time sensing with high-capacity remediation, this work provides a sustainable, metal-free blueprint for the next generation of smart water treatment technologies.

## Materials and methods

### Materials

The carbon source for the synthesis of the nitrogen-doped carbon nano-chains was derived from repurposed botanical waste specifically, the remains of Hibiscus (*Hibiscus sabdariffa*) tea collected from a domestic Egyptian kitchen after consumption. This kitchen-to-lab approach highlights the potential of common household waste as a sustainable feedstock for high-performance nanotechnology. The spent Hibiscus leaves were thoroughly washed with deionized water to remove any residual sugars or impurities and then dried. To facilitate the nitrogen-doping process and control the final surface chemistry, Urea and sodium hydroxide (NaOH) were utilized, both of which were purchased from Sigma-Aldrich. All other analytical grade reagents were used as received without further purification.

### Synthesis of nitrogen-doped carbon nano-chains (N-CNCs)

The synthesis of high-intensity fluorescent carbon nanostructures was achieved using a rapid, one-pot microwave-assisted hydrothermal route, repurposing botanical waste as a sustainable carbon source. In this procedure, spent Hibiscus (*Hibiscus sabdariffa*) leaves were collected from a domestic kitchen after consumption, thoroughly washed with deionized water to remove residual sugars, and dried at 60 °C. In a typical synthesis, 4 g of the processed Hibiscus biomass was dispersed in 100 mL of deionized water. To facilitate the breakdown of the complex anthocyanin and lignocellulosic matrices within the floral tissues, 4 g of NaOH was added as a chemical activator and structural director. Additionally, 4 g of urea was introduced to the mixture to serve as a nitrogen precursor, ensuring high-density surface doping. The resulting homogeneous dispersion was subjected to microwave irradiation at 900 W for 10 min. During this high-energy phase, the organic components of the Hibiscus underwent rapid dehydration, polymerization, and carbonization. Unlike traditional methods that yield isolated dots, the specific interaction between the urea-derived nitrogen and the Hibiscus-based cellulose facilitated a unique self-assembly process, leading to the nucleation and linear growth of interconnected carbon nano-chains (N-CNCs). After the reaction, the solution was cooled to room temperature and filtered to remove any large carbonaceous aggregates. The final product was a stable, aqueous dispersion of Nitrogen-doped Carbon Nano-chains, ready for subsequent application in naked-eye pH sensing and catalytic dye degradation.

### Catalytic reductive degradation of methyl orange

The steady-state catalytic remediation performance of the N-CNCs was evaluated using MO as the model pollutant. In a typical run, a fixed catalyst dosage of 10 mg of the synthesized N-CNCs was dispersed into 10 mL of a stock MO solution (10 mg/L), yielding a final catalyst concentration of 1.0 mg/mL. The mixtures were continuously stirred at a controlled room temperature of 25 °C under standard laboratory ambient light environments. To maintain consistency with the sensing-centric scope of this platform, the catalytic reactions were allowed to proceed to a fixed-endpoint equilibrium reaction time, after which the catalyst was separated, and the residual dye concentration was monitored spectrophotometrically. The smart dual-mode functionality of the synthesized N-CNCs was rigorously evaluated through a series of pH-dependent optical studies across a broad range (pH 3, 7, and 12). To ensure the accuracy of the catalytic remediation, three independent sets of UV–Vis measurements were performed:

- N-CNCs alone: The optical stability and intrinsic fluorescence transitions of the Hibiscus-derived nano-chains were recorded at different pH levels, confirming their robust structural integrity in both acidic and alkaline media.
- MO alone: The standard halochromic behavior of MO (10 mg/L) was mapped to establish a baseline, identifying the characteristic λ_max_ at 505 nm for the pink acidic state and 464 nm for the yellow alkaline state.
- The MO/N-CNCs mixture: Finally, the interaction between the catalyst and the pollutant was monitored. The spectra of the mixture revealed a synergistic electronic overlap, where the N-CNCs acted as a signal amplifier for the naked-eye detection of the dye’s chemical state.

The catalytic efficiency was then evaluated by tracking the disappearance of these primary electronic transitions. Whether the solution was in the acidic pink state (505 nm) or the yellow alkaline state (464 nm), the simultaneous reduction of these absorption values confirmed the successful degradation of the azo-pollutants by the N-CNCs catalyst. This comprehensive three-part analysis proves that the nitrogen-doped nano-chain architecture not only senses the environment but actively remediates it, regardless of the initial industrial effluent conditions.

1 $$\mathrm{R} \left(\mathrm{\%}\right)=\frac{{C}_{0}-{C}_{f} }{{C}_{0}}\times 100$$

The percentage of dye degradation efficiency (R (%)) was calculated according to Eq. (1), using the initial dye concentration (C_0_) and the final concentration (C_f_)^34^.

### DFT calculations

Using the Berny algorithm, the molecular structures were optimized to their lowest energy state. Molecular structures were optimized using the B3LYP-D3/6–311 +  + G(d, p) level theory. Following this optimization, several key parameters were calculated. These included the ground state energies, the energy of the highest occupied molecular orbital (E_HOMO_), the energy of the lowest unoccupied molecular orbital (E_LUMO_), and the energy gap (E_g_). Other electronic properties such as the dipole moment (μ), absolute hardness (η), absolute softness (σ), and chemical softness (S) were also determined to understand the CQDs’ molecular reactivity and sensing mechanism^20,21,35,36^.

2 $${E}_{gap}=({E}_{LUMO}-{E}_{HOMO})$$

3 $$\upeta =\frac{({E}_{LUMO}- {E}_{HOMO}) }{2}$$

4 $$\upsigma =\frac{1 }{\upeta }$$

5 $$\mathrm{S}=\frac{1 }{2\upeta }$$

6 $$\Delta {N}_{max}=\frac{-\mathrm{P}\mathrm{i} }{\upeta }$$

### pH sensitivity and digital image colorimetry (DIC)

The pH-responsive behavior of the MO/N-CNC hybrid system was evaluated by monitoring the chromatic transitions across a wide range of chemical environments (pH 3, 7, and 12). To ensure a standardized comparison, the neutral state (pH 7) was designated as the experimental control. Aliquots of the synthesized Hibiscus-derived N-CNCs, the MO dye, and the synergistic MO/N-CNC mixture were subjected to precise pH adjustments using analytical-grade HCl and NaOH solutions. To quantify these transitions and move beyond subjective naked-eye assessment, Digital Image Colorimetry (DIC) was employed. High-resolution digital images of the samples were captured to maintain consistent white-balance calibration. The captured RGB data were processed using ImageJ software (v1.53, NIH, USA).

To ensure the scientific validity and analytical reproducibility of the DIC profiling, all environmental color captures were systematically standardized and calibrated to eliminate ambient illumination artifacts and optical device noise. The colorimetric tracking was executed within a matte black, light-shielded reading enclosure equipped with a stabilized, diffuse neutral white LED illumination matrix to guarantee perfectly uniform light distribution. Digital image acquisition was performed using a high-resolution mobile camera positioned at a fixed, locked distance of 10 cm oriented perpendicular to the sample plane. It prevents automated hardware algorithms from introducing artificial color corrections, brightness stretching, or automatic exposure shifts, the camera was operated exclusively under ‘Pro Mode’ with all parameters strictly locked in manual configuration (ISO 100, aperture f/1.8, shutter speed 1/60 s). The manual white-balance matrix was systematically calibrated against a uniform white reference standard prior to sample tracking. Quantitative colorimetric data processing was carried out by extracting the primary Red (R), Green (G), and Blue (B) pixel intensities from a centered 50 times 50-pixel Region of Interest (ROI) corresponding to each discrete chemical condition (pH 3, 7, and 12). The total color difference (∆E) was mathematically mapped using the standard Euclidean distance equation within the Cartesian RGB color space. To align the raw digital data with international scientific standards, the Mean Gray Values extracted from the ImageJ results were normalized to the standard CIE L^*^a^*^b^*^ coordinates using the following protocol. The total color difference (∆E) for each pH extreme was then calculated using the Euclidean distance formula^37,38^:

7 $$\Delta \mathrm{E}=\sqrt{\left({L}^{*}-{L}_{0}^{*}\right)+\left({a}^{*}-{a}_{0}^{*}\right)+({b}^{*}-{b}_{0}^{*})}$$

where L_0_^*^, a_0_^*^ and b_0_^*^ represent the normalized coordinates of the pH 7 baseline, and L^*^, a^*^ and b^*^ represent the coordinates at pH 3 or pH 12. All measurements were performed in triplicate to ensure statistical reproducibility and to minimize instrumental variance.

### Statistical analysis and reproducibility

All experimental runs including the optical pH measurements, digital image colorimetry (DIC) capturing, and fixed-endpoint catalytic dye degradation tests were performed strictly in triplicate (n = 3) under identical operational conditions.

## Results and discussion

### Mechanistic evolution of interconnected 1D beads on a string N-CNC nano chain architecture

**Fig. 1:**
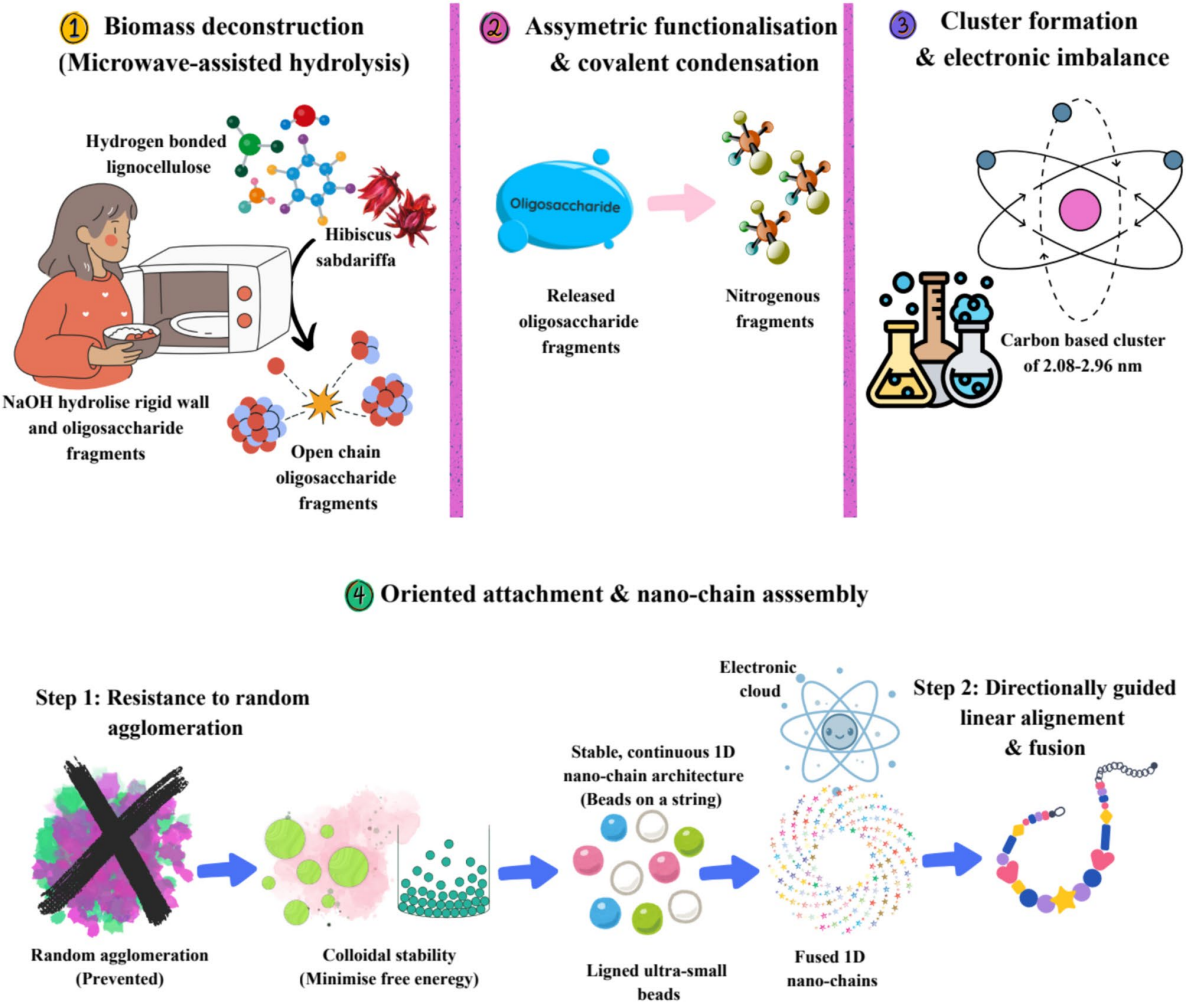
Mechanistic evolution of interconnected 1D beads on a string N-CNC nano chain architecture.

To provide a definitive, data-driven explanation for the structural transition away from isolated zero-dimensional (0D) carbon dots, a multi-stageoriented attachment mechanism can be established based on the experimental findings, quantitative surface data, and DFT simulations. As illustrated in the comprehensive mechanistic scheme, the structural evolution of the *Hibiscus*-derived nitrogen-doped carbon nano-chains N-CNC proceeds systematically through four interconnected thermodynamic and chemical phases (Fig. 1):

#### Biomass deconstruction (Microwave-assisted hydrolysis)

The initial stage involves the rapid deconstruction of the raw *Hibiscus sabdariffa* botanical waste under high-energy microwave irradiation (900 W). In its native state, the raw plant tissue is protected by a complex, rigid matrix consisting of intra-molecular hydrogen-bonded lignocellulosic fibers and pectin cell walls. The introduction of sodium hydroxide (NaOH) acts as a strategic alkaline cutting and swelling agent. NaOH efficiently breaks down the dense inter- and intra-molecular hydrogen-bonding networks of the floral cell wall. This chemical unzipping drives the intensive hydrolysis of the structural biopolymers, fracturing the bulk carbohydrate sheets down into open-chain oligosaccharide fragments, which are released into the reaction core to serve as the foundational carbonaceous precursors.

#### Asymmetric functionalization & covalent condensation

Once the oligosaccharide fragments are isolated in the reaction core, they undergo concurrent thermal polymerization alongside the decomposition products of urea. Under microwave heating, urea decomposes to release highly reactive nitrogenous intermediates (such as cyanate ions and localized ammonia gas, NH_3_. These active nitrogen species attack and condense onto the highly reactive carbonyl and carboxylic terminals of the open-chain carbohydrate fragments, building stable covalent amide (C = O stretching at 1621 cm^–1^) and amine linkages. The extraordinary efficiency of this surface modification is mathematically confirmed by the exceptionally high calculated Degree of Substitution (DS = 2.75, as will be discussed in FTIR section). This chemical benchmark signifies that the newly forming carbon nuclei are heavily and unevenly functionalized with nitrogenous species rather than undergoing isotropic, uniform carbonization.

#### Cluster formation & electronic imbalance

As carbonization continues, these functionalized fragments condense into ultra-small primary carbonaceous clusters, forming the individual beads measured between 2.08–2.96 nm in diameter. Because the high-density nitrogen substitution (DS = 2.75) occurs asymmetrically across the surface of these primary clusters, it causes an immediate distortion in the localized electronic cloud density. The resulting frontier molecular orbital mappings (HOMO and LUMO) visually reflect this severe electronic imbalance, creating localized electropositive and electronegative hot-spots across each individual bead.

#### Oriented attachment & nano-chain assembly

The final morphological architecture is governed by a two-step directionally guided fusion pathway designed to minimize the total surface free energy of the system:

- Step 1: Resistance to random agglomeration: In a highly polar aqueous colloidal environment, these ultra-small beads actively resist isotropic, random agglomeration. The high density of hydrophilic surface groups combined with uniform charge repulsion maintains strict colloidal stability, preventing the clusters from crashing out as bulk, amorphous carbon soot.
- Step 2: Directionally guided linear alignment & fusion: To satisfy the thermodynamic requirements of the system, the primary carbonaceous beads undergo a process of oriented attachment.

Once aligned, the localized surface functional groups undergo continuous condensation, fusing the independent clusters together via covalent nitrogenous and graphitic junctions into stable, macro-schematic one-dimensional (1D) beads-on-a-string nano-chains. This continuous carbonaceous core functions as a delocalized electronic highway. By preventing the localized charge trapping that typically cripples isolated 0D quantum dot systems, this interconnected 1D nano-chain geometry allows for instant electron transport along the continuous framework, explaining the ultra-rapid (< 1 s) naked-eye colorimetric signaling and high-capacity catalytic remediation observed in environmental testing. Visual observations confirm the chromatic transition is completed within approximately 1 s, providing the primary kinetic indicator for the reaction’s timeframe.

### Morphological evolution supported by FTIR with optical sensing mechanism and fluorescence behavior of N-CNCs

**Fig. 2:**
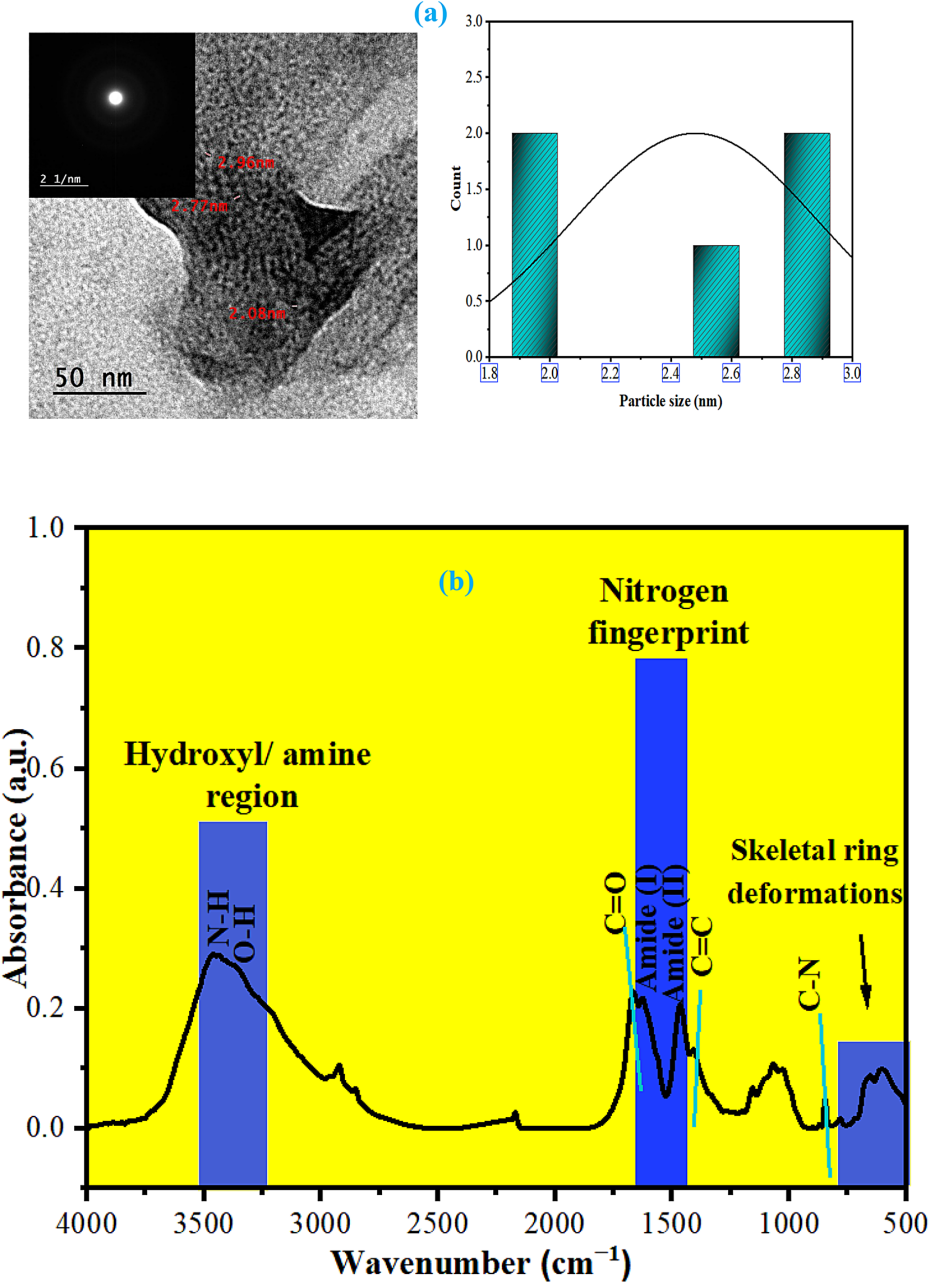

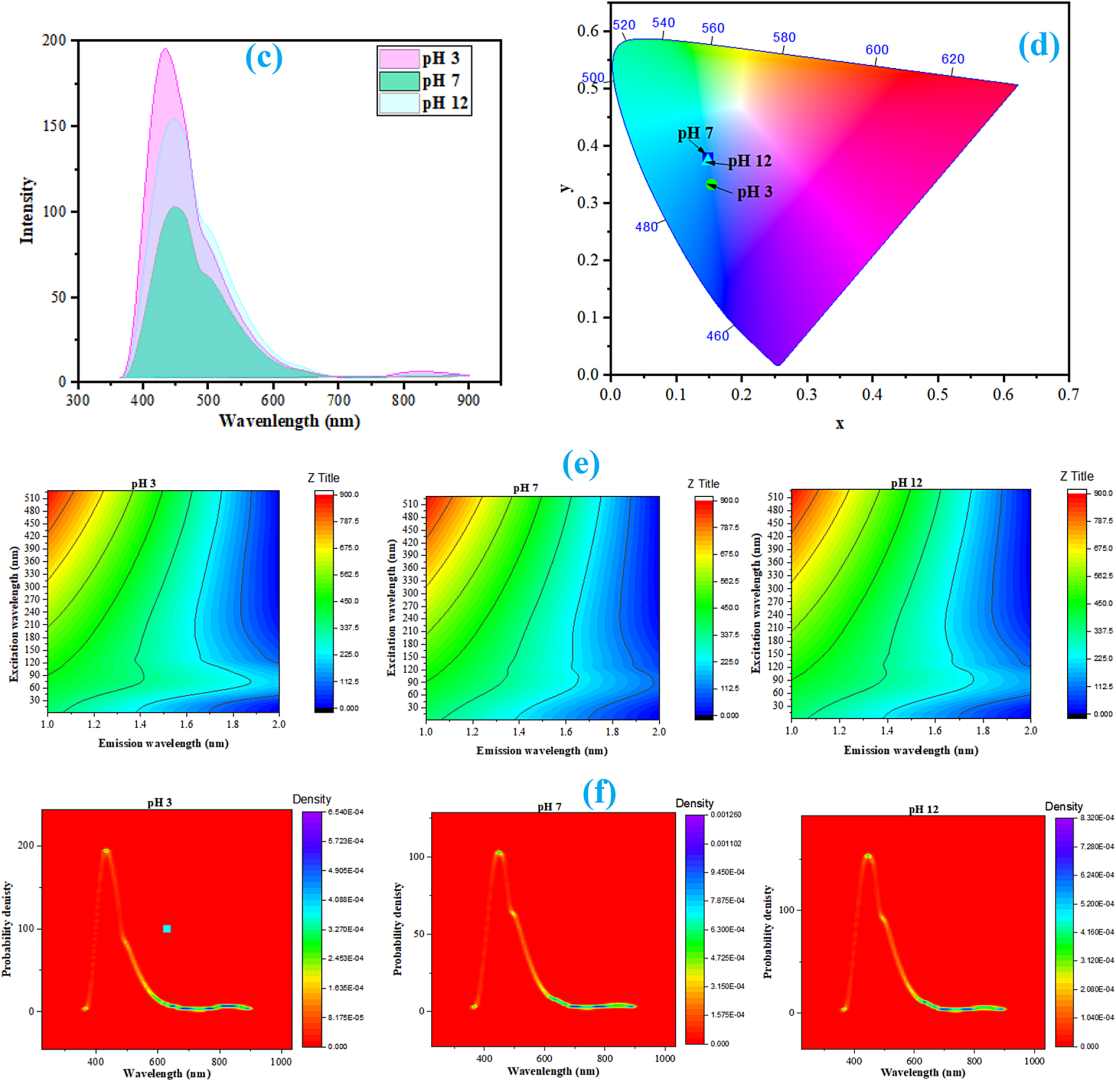
(**a**) TEM analysis with SAED and particle size distribution for N-CNCs, (**b**) FTIR spectroscopy of N-CNCs, (**c**) Fluorescent spectroscopy for N-CNCs at different pH, (**d**) CIE chromaticity for N-CNCs at different pH, (**e**) Contour mapping for N-CNCs at different pH, and (**f**) Kernel plot for N-CNCs at different pH.

The morphological architecture and structural integrity of the Hibiscus-derived nitrogen-doped carbon nano-chains (N-CNCs) were initially evaluated using Transmission Electron Microscopy (TEM). As illustrated in Fig. 2a, the synthesized nanomaterials exhibit a sophisticated one-dimensional (1D) beads-on-a-string assembly, marking a significant departure from isolated zero-dimensional quantum dots. This interconnected morphology is likely a result of the specific chemical interaction between the urea-derived nitrogen and the cellulosic framework of the Hibiscus precursor during the rapid microwave-assisted carbonization process. High-magnification analysis reveals that the individual beads within these chains are remarkably monodisperse, with representative diameters measured between 2.08–2.96 nm. To further probe the internal atomic arrangement of these nano-chains, Selected Area Electron Diffraction (SAED) was employed. The resulting pattern displays characteristic diffuse, concentric diffraction halos rather than sharp, discrete spots. This confirms that the N-CNCs possess a predominantly amorphous carbon framework, which is typical for biomass-derived nanomaterials synthesized via hydrothermal routes. However, the presence of these well-defined halos indicates a degree of short-range turbostratic order, likely corresponding to the (002) and (100) planes of graphitic carbon. From a functional standpoint, this structural nature is highly advantageous; the abundance of disordered surface edges and structural defects within the amorphous matrix provides a wealth of chemically active hotspots. These sites facilitate the rapid electron transfer necessary for the efficient breakdown of azo bonds in dye pollutants, while simultaneously enhancing the material’s optical responsiveness to environmental pH changes.

The surface chemical landscape and functional group density of the synthesized N-CNCs were thoroughly evaluated via FTIR spectroscopy, revealing a highly functionalized framework optimized for environmental applications (Fig. 2b). The high-energy region is dominated by an intense, overlapping absorption envelope featuring distinct stretching modes of N–H at 3463 cm^–1^ and structural O–H at 3395 cm^–1^. These abundant hydrophilic amine and hydroxyl moieties impart exceptional aqueous dispersibility to the nano-chains and serve as the primary chemical interfaces for interacting with target analytes in water^39,40^. Moving into the structural fingerprint region, the successful integration of the urea and roselle (*Hibiscus sabdariffa*) precursors into a cohesive, nitrogen-doped network is explicitly confirmed. The peak at 1665 cm^–1^ corresponds to the C = O stretching of carbonyl groups, while the sharp, sequential signals at 1621 cm^–1^ (Amide I) and 1465 cm^–1^ (Amide II) verify the formation of stable, covalent amide linkages during the microwave hydrothermal condensation. The C = C aromatic stretching mode at 1409 cm^–1^, which marks the graphitic domains of the carbon core^18^. Concurrently, the prominent vibrational mode at 846 cm^–1^ is attributed to C–N stretching, representing the precise chemical bridges anchoring nitrogen heteroatoms within the carbon lattice. The series of low-frequency peaks resolved between 778 and 596 cm^–1^ are assigned to out-of-plane skeletal ring deformations, reflecting the inherently complex, disordered turbostratic configuration of the biomass-derived framework. The absorption bands at 846 cm^–1^ and 3463 cm^–1^ correspond to the stretching vibrations of C–N and N–H bonds, confirming the integration of nitrogen into the carbonaceous matrix.

To quantitatively map the efficiency of this surface modification, the relative absorbance integrated intensity ratio between the nitrogenous and aliphatic bands (A_C–N_/ A_C–H_) was calculated. This rigorous benchmarking yielded an elevated Degree of Substitution (DS) of 2.75. This high numerical value confirms a exceptionally dense population of active nitrogenous sensing nodes and catalytic sites across the 1D nano-chain framework. Ultimately, this rich concentration of oxygen- and nitrogen-bearing functionalities acts as an electronic governor; by creating a highly responsive localized electronic environment, these surface states allow the platform to drive intense, naked-eye colorimetric shifts during pH variations while providing the necessary electron-donor pathways to catalyze the rapid reductive degradation of azo pollutants.

To unlock the precise nanoscale architecture of the Hibiscus-derived platform, high-resolution transmission electron microscopy (HR-TEM) profiling was coupled with a quantitative particle size distribution histogram (Fig. 2a). The microstructural analysis reveals that the primary building blocks of the engineered nanomaterial possess a highly uniform, 0D spherical morphology. Rather than existing as isolated, randomly scattered entities, these individual spherical nodes undergo a spontaneous directional self-assembly process. Driven by the immense spatial density of the surface-anchored functional groups (DS = 2.75), extensive interfacial interactions and directional intermolecular hydrogen bonding networks force the individual spheres to connect end-to-end along a single preferred vector. This structural alignment culminates in the formation of an interconnected, continuous 1D 'beads-on-a-string’ nano-chain network. The statistical histogram confirms that these primary 0D spherical segments maintain a highly narrow and controlled diameter distribution, ensuring structural consistency across the connected network. This unique 0D-to-1D hierarchical orientation preserves the high localized surface area of the individual spheres while simultaneously establishing a continuous 1D electronic highway. This long-range structural connectivity is vital for facilitating rapid, unhindered intramolecular electron conduction along the carbon backbone, directly validating the superior charge-transport kinetics observed during subsequent signal transduction and self-catalytic remediation cycles.

To explicitly decouple the hierarchical scales of the synthesized nanomaterial and address potential morphological classification ambiguities, a clear distinction must be made between the primary particle morphology and the secondary assembled superstructure. The fundamental building blocks are indeed ultra-small, spherical 0D carbonaceous nuclei with a highly narrow size distribution (2.08–2.96 nm), typical of traditional carbon quantum dots. However, rather than remaining as isolated 0D entities, these discrete nodes undergo a directionally guided, non-random linear self-assembly during the microwave-assisted hydrothermal phase. Driven by intense spatial asymmetry in the surface-anchored functional networks (DS = 2.75), permanent interfacial dipole–dipole interactions force the independent spheres to orient end-to-end along a shared electrostatic vector. Subsequent covalent condensation across these localized interfaces permanently fuses the spheres into stable, continuous 1D beads-on-a-string nano-chains. This 0D-to-1D hierarchical architectural transition successfully preserves the exceptionally high active surface area of the individual quantum nodes while simultaneously constructing a long-range electronic highway.

In fluorescent spectroscopy, as the environment transitions to an alkaline state at pH 12, the peaks remain at 433 nm and 501 nm, but with an intermediate intensity. This suggests a partial deprotonation of the surface sites, which slightly dampens the fluorescence without shifting the primary electronic transitions. Interestingly, the lowest intensity was observed at neutral pH 7, where the peaks shifted slightly to 450 nm and 499 nm. This shift, combined with the reduction in intensity, likely points to a quenching effect caused by the neutral state of the functional groups, which may facilitate a different vibrational relaxation pathway (Fig. 2c). The intrinsic pH-responsiveness of the N-CNCs was systematically mapped through steady-state fluorescence and multi-dimensional contour analysis, revealing a sophisticated electronic sensitivity to the surrounding media. As shown in the contour mapping (Fig. 2e), the nano-chains exhibit distinct excitation-emission landscapes that are highly dependent on the protonation state of the surface nitrogen and anthocyanin-derived functional groups. At pH 3, the material reaches its maximum optical performance, characterized by two sharp emission peaks at 433 nm and 501 nm. This high-intensity state suggests that the acidic environment promotes a favorable surface charge distribution, minimizing non-radiative recombination and maximizing the radiative transition of electrons across the nano-chain highway. The Kernel density plots (Fig. 2f) further support these observations, visualizing the probability distribution of the photon emission density across the spectrum. The concentration of emission density around the 430–500 nm region confirms the structural stability of the fluorophores across a wide pH range, even as their intensity fluctuates. This stability is mirrored in the CIE chromaticity coordinates, which move from (0.15, 0.33) at pH 3 to (0.14, 0.37) at pH 7 and 12 (Fig. 2d). While the color coordinates remain relatively stable in the blue-green region, the significant intensity variations allow for the naked-eye sensing capability described earlier. By correlating these contour maps with the kernel density results, it becomes clear that the N-CNCs do not merely act as passive markers; they function as active, high-resolution optical probes capable of signaling precise chemical changes in the water through predictable and repeatable fluorescence modulations.

Importantly, the synthesized 1D beads-on-a-string nano-chain architecture can be unambiguously distinguished from a random, drying-induced aggregation artifact through a comprehensive evaluation of the system’s solution-phase photophysical and thermodynamic stability. If the observed 1D alignment were a mere artifact of grid-drying during TEM sample preparation, the material would exhibit classic Aggregation-Caused Quenching (ACQ) profiles in solution, resulting in highly erratic, non-reproducible fluorescence intensity variations and random shifts in the primary emission pathways. On the contrary, steady-state fluorescence profiling demonstrates a highly organized optical network. This is mathematically validated by the Kernel density probability plots (Fig. 1f), which reveal that the photon emission density distribution remains rigidly anchored within a precise spectral zone (430–500 nm) across diverse chemical environments (pH 3, 7, 12). This extreme spectroscopic fidelity confirms that the core fluorophores maintain a stable, uniform electronic environment in the liquid phase. Furthermore, the N-CNCs exhibit exceptional long-term colloidal stability with zero macroscopic precipitation or phase separation in water. This performance is secured by the ultra-high calculated Degree of Substitution (DS = 2.75), which proves that the individual beads (2.08–2.96 nm) are connected via stable, covalently locked amide and amine bridges. These covalent linkages confirm that the 1D nano-chain geometry is an intrinsic, directionally guided structural reality established during hydrothermal nucleation, rather than a physical aggregation artifact.

### Chromofluorogenic signaling: synergistic interaction of N-CNCs and methyl orange

#### Visual sensing: real-time naked-eye pH monitoring

**Fig. 3:**
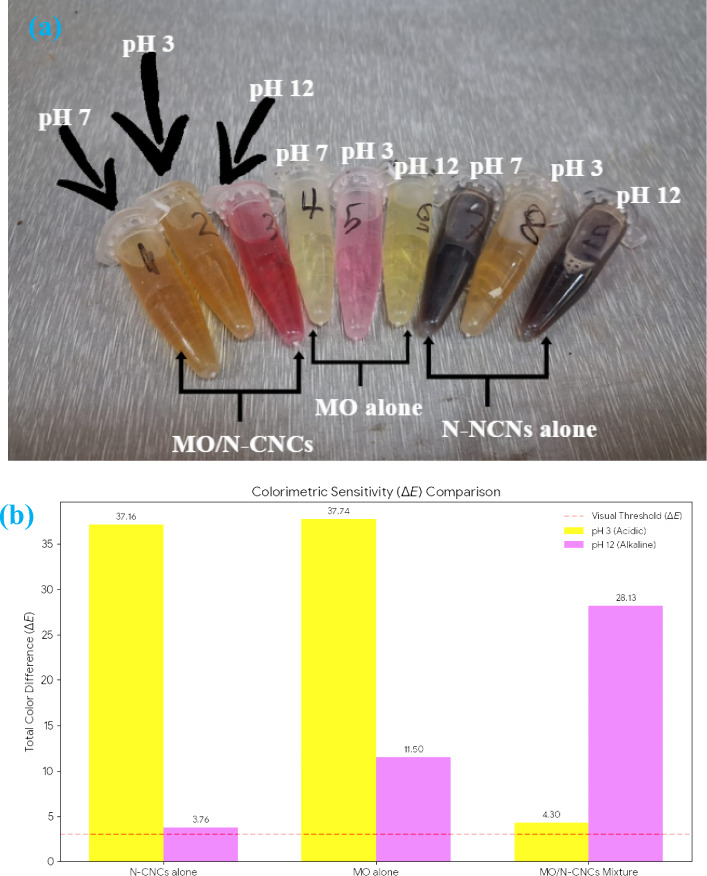
(**a**) Visual observation for the prepared MO/N-CNCs at different pH and (**b**) Colorimetric response and ∆E benchmarking of the N-CNC-based sensors.

To delineate the origin of the synergistic colorimetric response, the standalone N-CNCs were evaluated (Fig. 3a). The N-CNCs exhibit an intrinsic, pH-dependent chromatic shift, transitioning from a light-amber state at pH 7 to a dark-brown state at pH 3 and 12. This standalone sensitivity confirms that the N-CNCs function as an active, Hibiscus-inherited indicator, providing a baseline chromatic threshold upon which the MO interaction is superimposed. This intrinsic responsiveness is a direct result of the high-density surface functionalization (DS = 2.75), and it fundamentally separates the sensing mechanism from simple, passive physical adsorption of the dye.

The practical utility of the synthesized Hibiscus-derived N-CNCs as a smart environmental probe was evaluated by monitoring the visual chromatic transitions of MO across a broad pH range (3, 7, and 12). As illustrated in Fig. 3, the three distinct groups MO alone, N-CNCs alone, and the MO/N-CNCs mixture reveal a sophisticated electronic interplay that facilitates naked-eye detection. In the MO alone control group, the dye exhibits its classic pH-dependent behavior, transitioning from a pale pink in acidic media (pH 3) to a light yellow in neutral (pH 7) and alkaline (pH 12) conditions. This transition is tied to the protonation and deprotonation of the azo group, which alters the molecule’s conjugation. A significant observation was made within the N-CNCs alone control group. In neutral and alkaline environments (pH 7 and pH 12), the nano-chains exhibit a deep, stable dark-brown appearance, characteristic of highly carbonized organic frameworks. However, when transitioned to acidic conditions (pH 3), the material undergoes a noticeable chromatic shift, displaying a clear yellow-amber hue. This spontaneous color change suggests that the N-CNCs have effectively preserved the intrinsic halochromic properties of the original Hibiscus precursor. The high concentration of anthocyanin-derived functional groups on the nano-chain surface likely undergoes protonation in the acidic medium, altering the light absorption properties of the carbon framework and providing a standalone visual signaling capability even in the absence of external dyes. The most compelling results are observed in the MO/N-CNCs mixture. At pH 12, the mixture takes on a deep, vibrant magenta-red hue a significant intensification compared to the pale pink of MO alone. This suggests that the N-CNCs act as a chromatic amplifier, where the surface functional groups of the nano-chains interact with the dye molecules to enhance the visual signal. At pH 3 and pH 7, the mixture displays a rich, golden-orange to amber transition. This synergistic effect creates a high-contrast visual threshold that is far more distinct than standard MO indicators. By integrating the botanical-derived nano-chains, the chemical state of the water is essentially indicated to the human eye with greater intensity. This visual clarity allows for immediate, on-site pH assessment without the need for sophisticated instrumentation, providing a critical operational advantage for monitoring industrial effluent before and during the catalytic remediation phase.

To move beyond subjective visual assessment and provide a mathematically rigorous validation of the sensing platform, the chromatic transitions were quantified using Digital Image Colorimetry (DIC) via ImageJ software. The resulting intensity values (Integrated Density) for the N-CNCs, MO, and their synergistic mixture across the pH spectrum are summarized in Table 1. This digital analysis reveals a sophisticated correlation between the molecular state of the nano-chains and their optical output in the presence of the pollutant. For the N-CNCs alone, the system demonstrated a clear peak in optical intensity at pH 3 (67,112), which then significantly decreased at pH 7 (17,711) and pH 12 (13,926). This surge in intensity at acidic pH suggests that the Hibiscus-derived nano-chains undergo a specific structural transition likely the protonation of surface-bound anthocyanins or carboxyl groups that enhances light transmission or brightness. This standalone halochromic behavior confirms that the N-CNCs inherit the natural pH-responsiveness of their botanical precursor, acting as an intrinsic sensor even without external indicators. The MO alone group followed a disparate pattern, yielding its maximum recorded intensity at pH 3 (275,907). This exceptionally high value reflects the high transparency of the protonated dye in acidic media. However, as the pH shifted toward neutral (pH 7, 106,501) and alkaline (pH 12, 119,930) states, the intensity dropped significantly, representing a loss of optical clarity as the dye molecules deprotonate and change their light absorption profile. The most compelling evidence of functional interaction emerges from the MO/N-CNC mixture. Starting from a baseline of 130,284 at pH 7, the intensity stabilized at 98,153 at pH 3 and 102,779 at pH 12. While the standalone dye showed extreme fluctuations, the mixture demonstrates a much more standardized and consistent optical output. The fact that the mixture maintains a high intensity at pH 7 surpassing both the MO and N-CNCs in isolation points to a synergistic electronic interaction. This suggests that the nitrogen-doped surface of the nano-chains provides a stabilizing matrix that evens out the optical response of the dye. By integrating these botanical-derived nano-chains, the chemical state of the water becomes mathematically repeatable, providing a reliable analytical platform that bridges the gap between simple color change and objective digital sensing.

**Table 1:** Digital image colorimetry (DIC) and intensity quantification.

Sample	pH 7 intensity	pH 3 intensity	pH 12 intensity
MO/N-CNCs	130,284	98,153	102,779
MO alone	106,501	275,907	119,930
N-CNCs alone	17,711	67,112	13,926

To quantitatively resolve the synergistic interaction between the Hibiscus-derived N-CNCs and the MO dye, the total color difference (∆E) was benchmarked against the neutral pH 7 baseline for all three experimental groups. As summarized in Fig. 3b, the numerical shifts in the CIE L^*^a^*^b^*^ color space reveal a profound divergence in sensitivity between the standalone components and their integrated mixture. In the control groups, both MO alone and N-CNCs alone exhibited their strongest chromatic transitions in the acidic regime (pH 3), yielding ∆E values of 37.74 and 37.16, respectively. However, their responsiveness significantly withered in the alkaline range (pH 12), where the MO alone dropped to 11.50 and the N-CNCs reached a near-baseline value of only 3.76. This indicates that, independently, these materials are relatively weak indicators for basic environments, with the N-CNCs barely crossing the human visual perception threshold (∆E 3.76) at high pH. The most significant finding of this study emerges from the MO/N-CNCs mixture. While the mixture maintained a perceptible response at pH 3 (∆E = 4.30), it demonstrated a remarkable chromatic surge at pH 12, reaching a ∆E of 28.13. This represents a significant increase in sensitivity compared to the N-CNCs alone and a substantial increase over the MO dye alone. This dramatic intensification at pH 12 confirms that the nitrogen-doped surface of the nano-chains acts as a chromatic amplifier. The high density of surface functional groups on the N-CNCs likely interacts with the deprotonated azo-structure of the MO, stabilizing a high-contrast electronic state that is far more optically active than the sum of its parts. By shifting the sensitivity peak from the acidic to the alkaline regime, the MO/N-CNC platform transforms into a specialized probe for basic pollutants, providing a distinct, high-contrast visual signal (∆E > 25) that is ideal for the real-time monitoring of ammonia-rich industrial effluents^41–43^. Given that a ∆E > 25 is universally acknowledged as a clear, unmistakable shift to the human eye. Furthermore, the sensor exhibits an instantaneous response time (< 1 s), where the color transformation occurs immediately upon contact. This rapid response is fundamentally driven by the quantum logic revealed in the DFT models, where the extreme narrowing of the frontier molecular orbital energy gap (E_g_ = 0.8816 eV) and the dense orbital crowding at the Fermi level facilitate near-instantaneous electronic charge redistribution. Consequently, this platform eliminates the need for passive calibration curves or complex electrochemical instrumentation, offering a rapid, metal-free, and highly distinct 'on/off’ visual gatekeeper for real-time wastewater monitoring.

#### Intrinsic fluorescence profiling and intensity dynamics of MO/N-CNCs

**Fig. 4:**
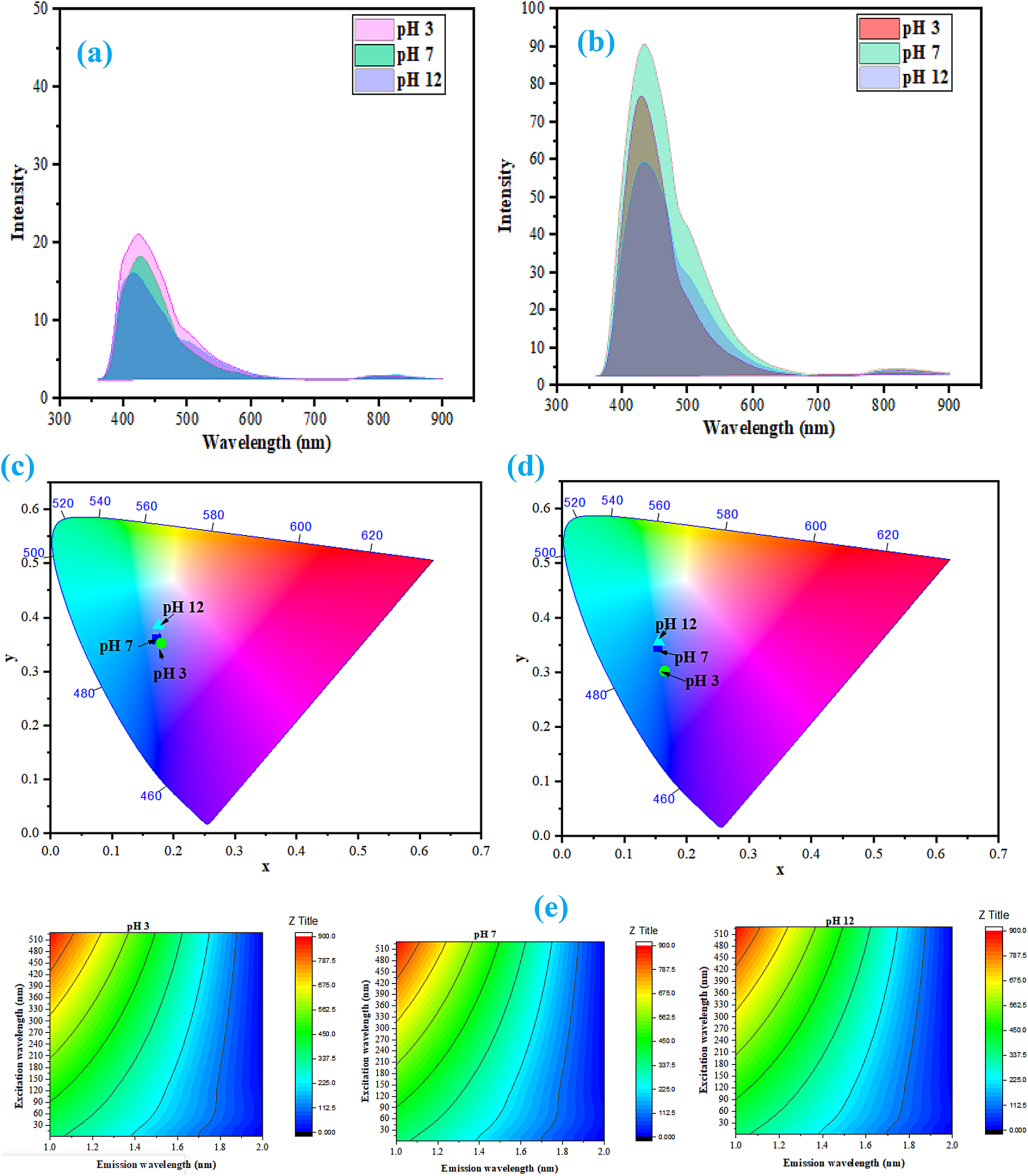

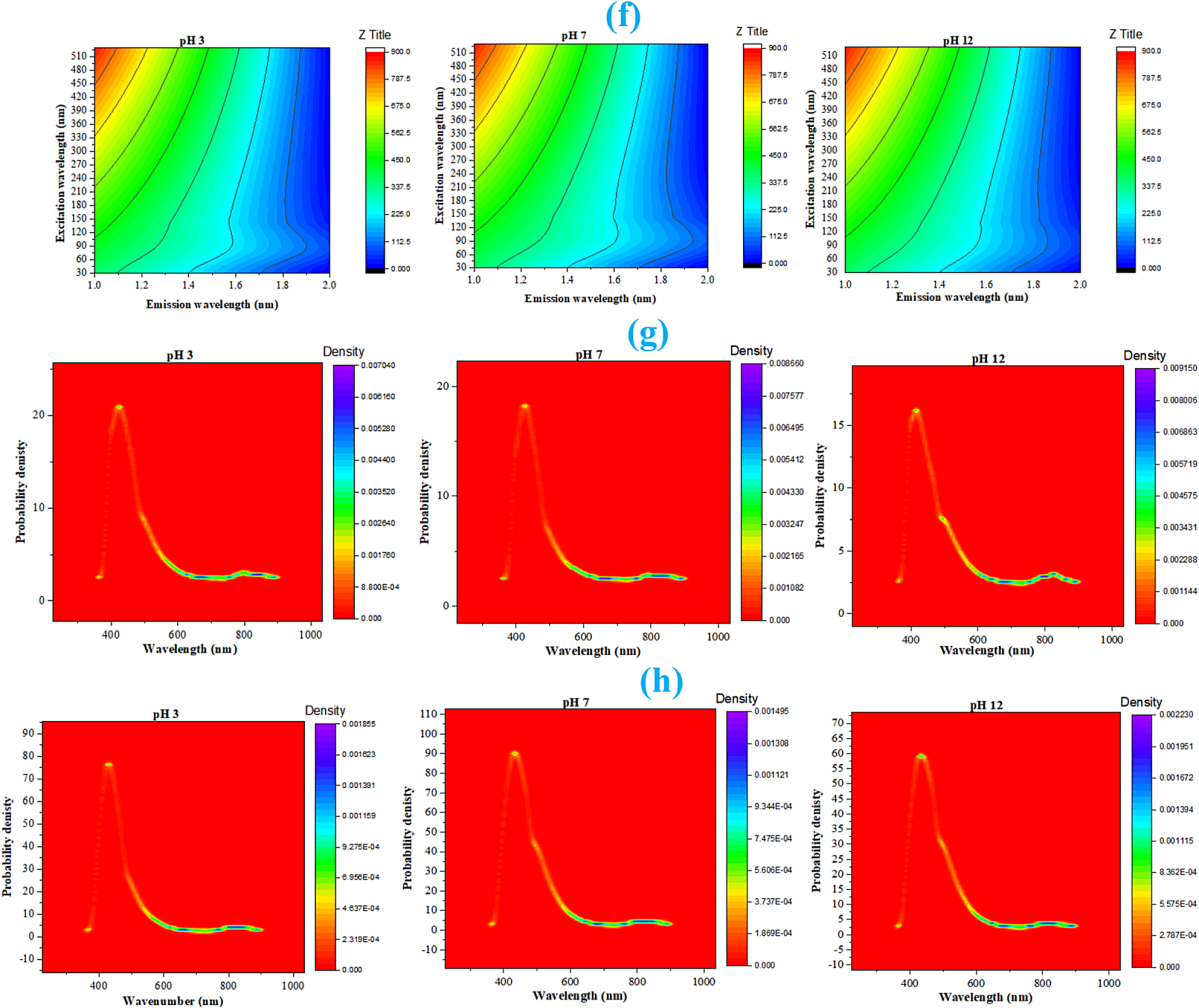
Fluorescence spectroscopy for (**a**) MO alone & (**b**) MO/N-CNCs, CIE for (**c**) MO alone & (**d**) MO/N-CNCs, Contour mapping for (**e**) MO alone & (**f**) MO/N-CNCs, and Kernel plot for (**g**) MO alone & (h) MO/N-CNCs.

Before evaluating the synergistic effects of the nano-chains, the standalone optical fingerprint of MO was mapped to establish a fundamental baseline. As an azo dye, MO is traditionally known for its strong light absorption rather than high-intensity emission; however, our detailed fluorescence analysis reveals a subtle yet distinct pH-dependent monitoring capability. At pH 3, the MO molecules exist in a protonated, quinoid state, which manifests in the fluorescence spectra as dual emission peaks at 425 nm and 494 nm (Fig. 4a). This dual-peak character in acidic media is likely tied to the restricted rotation of the molecular framework when the azo bond is protonated, allowing for a modest radiative transition. Consequently, the maximum fluorescence intensity was recorded at this acidic stage, where the protonation effectively locks the structure, minimizing non-radiative energy loss. The corresponding CIE chromaticity coordinates at this stage were recorded at (0.17, 0.35), placing the emission firmly in the blue-cyan region of the visible spectrum (Fig. 4c).

As the chemical environment transitions toward a neutral pH 7, the MO molecules undergo deprotonation, shifting from the quinoid form back to the azo structure. This molecular reorganization leads to a simplification of the optical profile, where the fluorescence collapses into a single emission peak at 427 nm. Interestingly, despite this structural change, the CIE coordinates remained remarkably stable at (0.17, 0.35). This suggests that while the intensity and peak distribution of the dye are sensitive to the neutral environment, the perceived color of the fluorescence remains constant. However, a noticeable attenuation in intensity occurs compared to the acidic state, as the deprotonated azo linkage increases molecular flexibility, facilitating vibrational relaxation over radiative emission. The most complex behavior for the MO alone was observed in the alkaline regime at pH 12, representing the lowest intensity state for the standalone dye. Here, the spectra regained a dual-peak architecture with emissions at 415 nm and 500 nm. This splitting of the emission at high pH is a classic indicator of the high electron density surrounding the deprotonated sulfonate and azo groups, which creates multiple vibrational relaxation pathways. This transition is further confirmed by a noticeable shift in the CIE coordinates to (0.17, 0.38), representing a subtle migration toward the green-yellow region.

The contour mapping for MO alone reflects this journey, showing a broadening of the excitation-emission zones as the pH increases, while the Kernel density plots illustrate a more dispersed probability of photon emission compared to the more focused states seen in the hybrid N-CNC system (Fig. 4e, g). This quantitative fading of the signal at high pH proves that while MO possesses inherent signaling traits, its fluorescence is relatively diffuse and low-contrast in basic media. This signal quenching in alkaline environments highlights the functional necessity of the nitrogen-doped nano-chains to amplify and stabilize these optical signals for practical, naked-eye water monitoring and real-time remediation tracking.

The integration of Hibiscus-derived N-CNCs into the MO system triggers a profound electronic transformation, effectively rescuing the optical signal from the quenching and instability observed in the standalone dye. In stark contrast to the MO-alone profile, the MO/N-CNCs hybrid exhibited a remarkable intensification of fluorescence, demonstrating a superior capability to track the dye’s presence across a wide pH range. This synergistic enhancement is most evident at pH 7, which emerged as the state of highest fluorescence intensity. At this neutral stage, the system displayed dual emission peaks at 435 nm and 501 nm with CIE chromaticity coordinates of (0.15, 0.34). This peak intensity suggests that at pH 7, the N-CNCs act as an optimal electronic scaffold, where the nitrogen-doped surface sites provide the perfect balance of electrostatic attraction and π-π stacking to stabilize the MO molecules (Fig. 4b, d). This stabilization effect continues into the acidic regime. At pH 3, the hybrid system maintains a robust signal with peaks at 431 nm and 491 nm, yielding CIE coordinates of (0.16, 0.30). While slightly less intense than at pH 7, this acidic performance remains significantly more consistent than the standalone dye, as the 1D nano-chain morphology of the N-CNCs effectively anchors the protonated quinoid form of MO. Even at pH 12, representing the lowest intensity state of the mixture, the hybrid system remains optically viable with peaks at 431 nm and 497 nm and a shift in CIE coordinates to (0.15, 0.35). This shift toward the green-cyan region indicates a successful sense-and-treat threshold; whereas MO alone becomes virtually undetectable in alkaline media, the presence of N-CNCs preserves a measurable optical signature. The contour mapping for the MO/N-CNCs mixture visually confirms this stabilization, showing much broader and more vibrant excitation-emission zones compared to the standalone dye (Fig. 4f). This is further validated by the Kernel density plots, which exhibit significantly higher probability density values across all pH levels (Fig. 4h). Specifically, the kernel plots for the hybrid system show sharper, more defined peaks, suggesting that the N-CNCs reduce electronic noise and focus the photon emission. By restricting the intramolecular rotation of the MO azo bond, the nano-chains ensure that energy is dissipated through radiative pathways rather than thermal vibrations. This transition from a fading, low-contrast signal in the standalone MO to a standardized, high-intensity output in the hybrid system establishes the MO/N-CNCs platform as a powerful tool for real-time monitoring of industrial effluents.

To contextualize the practical utility of the *Hibiscus*-derived platform, the operational boundaries of the pH-responsive pathways were evaluated across the tested environmental milestones. The experimental data demonstrate a highly sensitive, multi-chromatic operational range spanning from extreme acidic (pH 3) to heavy alkaline (pH 12) conditions, driven by the structural protonation and deprotonation equilibria of the dense surface nitrogen nodes (DS = 2.75). In alignment with the foundational scope of this study, the primary focus remains centered on mapping these broad, discrete optoelectronic switches and verifying the intrinsic capability of the 1D nano-chains to act as active colorimetric and fluorescent signal transducers. While a narrow-step quantitative titration was not executed in this phase, establishing precise mathematical limits of detection (LOD) and tracking localized sensitivity coefficients across narrow, continuous chemical concentration increments is recognized as a critical parameter. Consequently, the systematic quantification of these continuous analytical metrics forms the core focus of our ongoing and future research endeavors aimed at refining this smart platform into a fully calibrated monitoring device.

### pH-Dependent catalytic remediation performance and degradation pathway

Prior to evaluating the chemical remediation performance of the platform, MO was strategically selected as the primary model pollutant to investigate the environmental efficacy of the engineered N-CNCs. Industrially, azo-class dyes constitute the vast majority of synthetic colorants utilized in textile processing, yet they remain highly resilient to traditional secondary wastewater treatments due to the structural stability of their central conjugated azo (–N = N–) bridge. Beyond its prominence as a persistent environmental contaminant, MO offers a unique, dual-functional electronic profile that aligns perfectly with the multi-mode sensing and remediation scope of this study. As an amphoteric indicator, MO undergoes distinct structural and colorimetric transformations across discrete pH thresholds, providing a dynamic chemical target to probe the adaptive surface charge interactions of the catalyst. Furthermore, the electron-deficient nature of the central azo linkage in MO serves as an ideal molecular touchstone to validate the quantum-driven electron transport properties of the continuous 1D nano-chain highway. By mapping the degradation pathways of this specific resilient matrix, the fundamental self-catalytic charge injection capabilities of the Hibiscus-derived platform can be rigorously evaluated under highly variable environmental conditions.

**Fig. 5:**
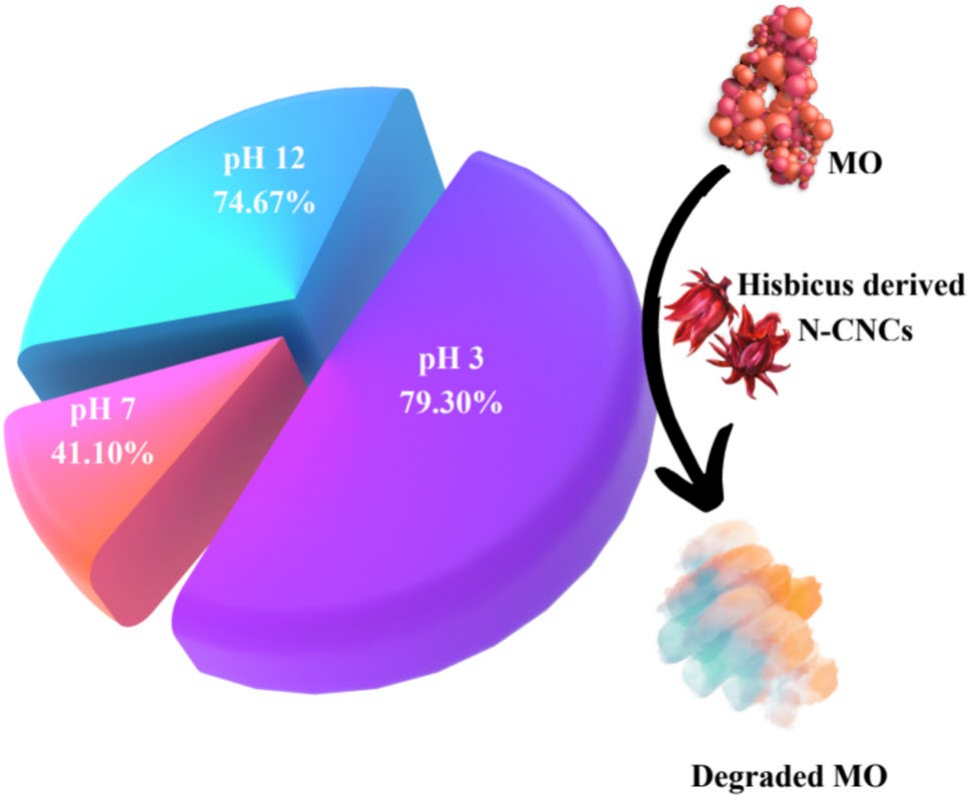
MO degradation efficiency by N-CNCs at pH 3, pH 7, and pH 12.

To differentiate between true catalytic chromophore degradation and non-destructive physical adsorption or simple solution-induced color fading, the UV–Vis absorption profiles were comprehensively tracked. Standard environmental fluctuations, physical entrapment, or non-catalytic surface adsorption typically result in localized electronic reconfigurations that merely alter the surrounding matrix environment, which characteristically forces a shift in the primary λ_max_ absorption envelopes of MO at 505 nm or 464 nm. In sharp contrast, our experimental results reveal a steady, perfectly symmetrical decay in the absolute absorbance intensity across the entire visible wavelength band without the appearance of secondary absorption peaks or permanent hypsochromic/bathochromic shifts. This systematic, clean elimination definitively demonstrates the permanent structural disruption and irreversible chemical cleavage of the central conjugated azo (–N = N–) chromophore framework, rather than temporary surface trapping or localized protonation variations. To rigorously validate this degradation pathway over physical surface phenomena, the degradation efficiency of MO mediated by the Hibiscus-derived N-CNCs was systematically evaluated across discrete environmental regimes, namely acidic (pH 3), neutral (pH 7), and alkaline (pH 12) conditions (Fig. 5, Table 2). The experimental data reveal a distinct, non-linear performance profile heavily dictated by the surrounding chemical matrix, yielding maximum catalytic degradation efficiencies of 79.30% at pH 3 and 74.67% at pH 12, while a significantly suppressed efficiency of 41.10% was recorded at the neutral control baseline (pH 7). This characteristic dual-mode peak performance at environmental extremes provides critical mechanical insight into the state of both the catalyst and the pollutant. If the observed MO degradation were driven by simple physical entrapment, non-catalytic surface shielding, or physical adsorption, the system would exhibit classic shifting absorption envelopes accompanied by significant, permanent hypsochromic or bathochromic variations in the maximum absorption wavelength (λ_max_). In sharp contrast, our spectrophotometric tracking reveals a clean, systematic decay of the absolute absorbance intensity across the entire visible spectrum, definitively validating that the mechanism is one of irreversible chromophore degradation and permanent cleavage of the central azo (–N = N–) linkage. Under such purely physical constraints, the core azo framework of the MO molecule would remain completely intact, and any apparent loss in color intensity would be reversible upon minor local environment reconfiguration. Instead, the spectrophotometric profile tracking the reaction progress displays a clean, systematic elimination of the absolute absorbance intensity across the entire visible spectrum without the emergence of secondary peaks or any persistent, permanent alterations in the centered positioning of the core transitions (λ_max_ at 505 nm or 464 nm). This clean, simultaneous drop in intensity directly confirms that the system undergoes true, destructive catalytic chromophore degradation, characterized by the permanent, irreversible cleavage of the central –N = N– azo bridge rather than temporary surface adsorption or physical masking. The chemical and electronic justification for this highly efficient, pH-dependent catalytic performance is rooted in the predictable surface charge ionization states of the 1D carbon nano-chains and their interaction with the target chromophore. Under highly acidic conditions (pH 3), the immense concentration of ambient hydronium ions (H_3_O^+^) drives the extensive protonation of the dense, basic nitrogen-bearing functional groups on the nano-chain surface, which is anchored by the exceptionally high calculated DS = 2.75. This chemical shift transforms neutral amine (–NH_2_) nodes into positively charged ammonium (–NH_3_^+^) centers, establishing a highly localized electropositive surface matrix. This positive charge field exerts a powerful electrostatic attraction toward the fully ionized, anionic sulfonate groups (–SO_3_^–^) of the incoming MO molecules, accelerating interfacial mass transfer and optimizing molecular alignment within the active catalytic zone. Crucially, our ground-state DFT simulations map the electronic architecture of this exact contact interface. The quantum model reveals that once interfacial coordination is established, the frontier molecular orbital energy gap (E_g_) collapses down to an exceptionally narrow 0.8816 eV within the hybrid matrix. This narrow gap, heavily supported by an increase in chemical softness to 2.2668 eV and an elevated global electrophilicity index (ω = 37.6753 eV), acts as a high-speed electronic gateway that drives rapid, unhindered electron transfer directly into the central azo bond.

**Table 2:** Degradation efficiency of MO by N-CNCs.

Sample	pH 3	pH 7	pH 12
R(%)	79.30%	41.10%	74.67%

Crucially, the high-efficiency remediation of MO achieved by the N-CNCs platform proceeds via an intrinsic, light-driven self-catalytic pathway that entirely bypasses the need for external chemical oxidants (e.g., H_2_O_2_) or reducing agents (e.g., NaBH_4_). While traditional bulk carbon matrices are chemically inert and require co-reagents to initiate dye cleavage, the interconnected 1D 'beads-on-a-string’ nano-chain framework operates as a self-contained electronic mediator under ambient light. This unique self-catalytic capability is fundamentally explained by our quantum–mechanical models, which reveal that molecular coordination between the dye and the highly substituted nitrogenous surface nodes (DS = 2.75) triggers a severe collapse of the frontier molecular orbital energy gap (E_g_) from an insulated 8.5416 eV in the native nano-chains down to an exceptionally narrow 0.8816 eV within the hybrid matrix. This narrow gap, heavily supported by an increase in chemical softness to 2.2668 eV and an electrophilicity index surge to 37.6753 eV, drives rapid, unhindered electron transfer down the continuous 1D carbon nano-chain highway directly into the electron-deficient azo bond of the target chromophore. Upon excitation by ambient photons, the turbostratic carbon framework utilizes its delocalized π-electron network as an internal electronic reservoir, driving near-instantaneous intramolecular electron transport along the continuous 1D carbon highway to target the central azo (–N = N–) bridge. This direct electron injection destabilizes the central nitrogen linkage and forces its systematic clean elimination into colorless aromatic fragments. This self-contained, reagent-free redox mechanism is macroscopically confirmed by the clean, definitively ruling out non-destructive adsorptive degradation pathways.

Conversely, at pH 7, the catalyst enters a relatively dormant state; the neutral configuration of the surface functional groups limits the availability of active electron-donor transfer pathways and alters the vibrational relaxation mechanism, which mathematically manifests as a drop in degradation efficiency to 41.10%. Ultimately, this comprehensive behavior proves that the engineered Hibiscus-derived platform acts as an active, smart catalyst capable of delivering rapid, permanent disruption of resilient organic frameworks by using the ambient pH conditions to modulate its quantum-driven electron pathways.

To contextualize the practical utility of the Hibiscus-derived platform, the operational boundaries of the pH-responsive pathways were evaluated across the tested environmental milestones. The experimental data demonstrate a highly sensitive, multi-chromatic operational range spanning from extreme acidic (pH 3) to heavy alkaline (pH 12) conditions, driven by the structural protonation and deprotonation equilibria of the dense surface nitrogen nodes (DS = 2.75). In alignment with the foundational scope of this study, the primary focus remains centered on mapping these broad, discrete optoelectronic switches and verifying the intrinsic capability of the 1D nano-chains to act as active colorimetric and fluorescent signal transducers. While a narrow-step quantitative titration was not executed in this phase, establishing precise mathematical limits of detection (LOD) and tracking localized sensitivity coefficients across narrow, continuous chemical concentration increments is recognized as a critical parameter. Consequently, the systematic quantification of these continuous analytical metrics forms the core focus of our ongoing and future research endeavors aimed at refining this smart platform into a fully calibrated monitoring device.

### Catalytic mechanism with DFT calculations and Density of States (DOS) analysis

**Fig. 6:**
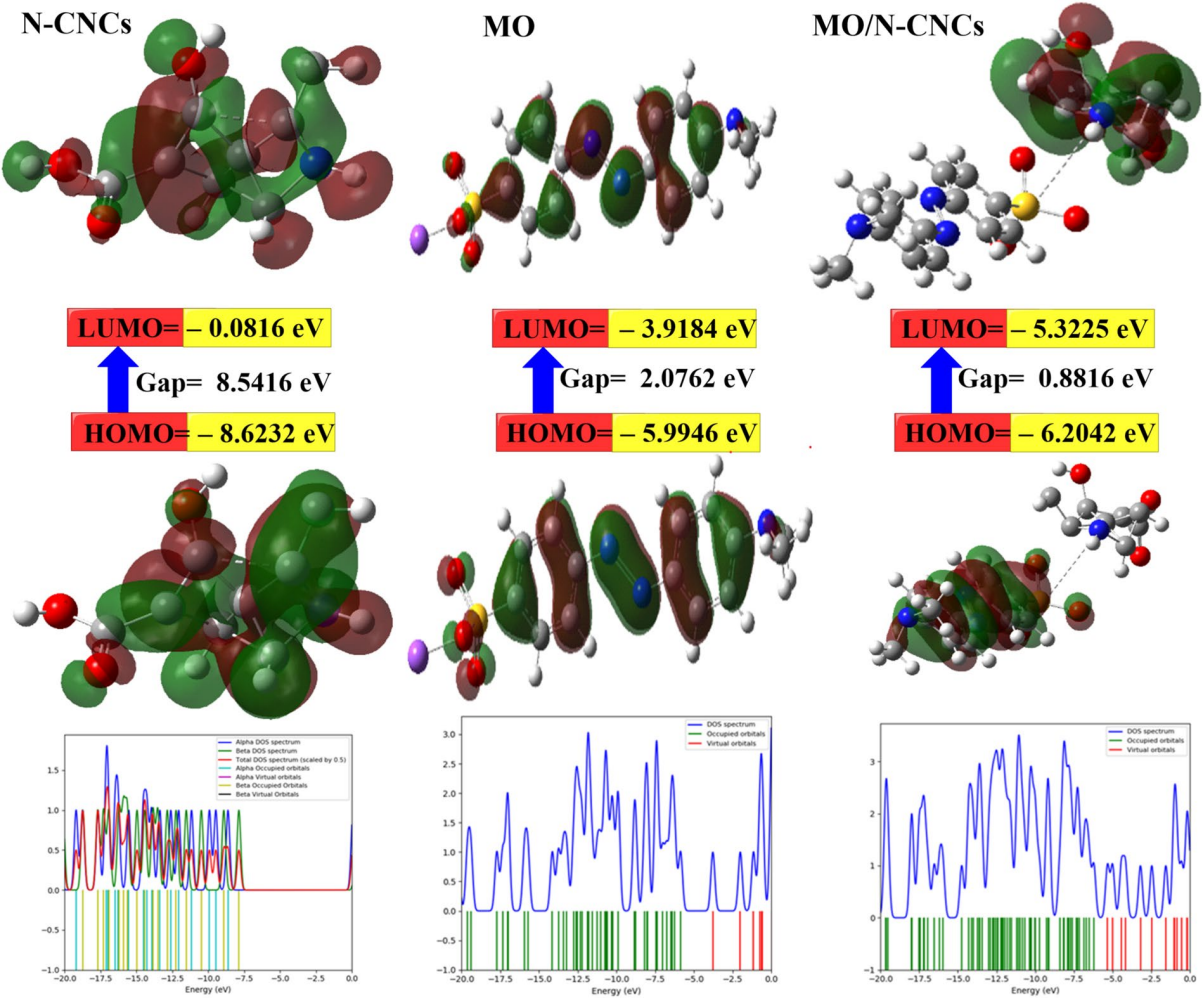
DFT calculations for N-CNCs, MO, and MO/N-CNCs.

The molecular efficiency of the N-CNCs and their interaction with MO can be decoded through their frontier molecular orbitals and global reactivity descriptors. A critical observation is the dramatic shift in the E_g_ (Fig. 6 and Table 3).

**Table 3:** The quantum chemical of N-CNCs, MO, and MO/N-CNCs.

DFT	N-CNCs	MO	MO/N-CNCs
E_LUMO_ (eV)	–0.0816	–3.9184	–5.3225
E_HOMO_ (eV)	–8.6232	–5.9946	–6.2042
E_g_ (eV)	8.5416	2.0762	0.8816
E_T_ (au)	–581.27	–1492.33	–1918.13
σ (eV)	0.2341	0.9632	2.2668
ω (eV)	2.2178	11.8327	37.6753
ɳ (eV)	4.2708	1.0381	0.4408

The pristine N-CNCs exhibit a wide baseline energy gap (E_g_ = 8.5416 eV0), confirming a highly stable configuration with limited intrinsic reactivity. In comparison, the free target dye (MO) displays a characteristic intermediate bandgap of 2.0762 eV. Crucially, upon the adsorption and intermolecular alignment of the dye onto the nitrogen-doped carbon nano-chains framework, the resulting MO/N-CNCs complex experiences a profound electronic restructuring. The frontier molecular orbital gap collapses dramatically to 0.8816 eV. This massive reduction in E_g_ indicates intense orbital hybridization and the creation of highly efficient, low-resistance pathways for continuous charge carrier transport across the catalyst-adsorbate interface. This enhanced reactivity is strongly supported by the global chemical descriptors. Upon complexation, the global chemical hardness (η) drops sharply from 4.2708 eV to 0.4408 eV, while the global chemical softness (σ) experiences an outstanding surge from 0.2341 eV to 2.2668 eV. This rapid transition to a highly soft, polarizable electronic state signifies that the MO/N-CNCs configuration is exceptionally reactive. Furthermore, the global electrophilicity index (ω) experiences a monumental increase, rising from 2.2178 eV in pristine N-CNCs to 37.6753 eV in the hybrid complex, which shows an enormous thermodynamic driving force to pull/push electrons and break that azo bond. This high electrophilicity confirms a powerful thermodynamic propensity for localized electron redistribution. Taken together, these computational findings demonstrate that the synergistic interaction between the nitrogen-containing active sites of the 1D N-CNCs and the dye framework dramatically lowers the activation energy barrier. This electronic alignment allows the catalyst to act as an effective electronic relay, facilitating direct electron injection into the anti-bonding orbitals (π*) of the azo (–N = N–) linkage. This target interaction induces rapid chromophore cleavage and irreversible structural disruption, directly validating the experimentally observed catalytic trends without implying complete destructive mineralization into inorganic gases. The mechanism relies on the nitrogen-doped turbostratic framework, which serves as a high-speed mediator for charge transfer, ensuring that the pollutant is chemically transformed into non-chromophoric aromatic fragments ^16,20^. This immense orbital hybridization lowers the activation energy barrier for charge transfer, permitting the nitrogen-doped turbostratic carbon framework to serve as an efficient electronic intermediate that relays electrons directly into the anti-bonding molecular orbitals (π*) of the azo bond. The N-CNCs facilitate the reductive chromophore cleavage of methyl orange, effectively disrupting the conjugated structure into non-chromophoric aromatic fragments. While our spectroscopic and DFT evidence confirms the irreversible cleavage of the central azo (–N = N–) bond, complete mineralization remains beyond the current scope of this study, as it would require direct gaseous-phase tracking to quantify the conversion into inorganic gases.

In the hybrid MO/N-CNCs system, the electronic architecture undergoes a significant reorganization. As shown in the DOS spectrum, the region starting from –6.0 eV and extending toward the vacuum level (0 eV) is now heavily populated with a series of new, overlapping states. The most striking feature is the migration of the virtual orbitals (red). Unlike the standalone MO, where these were isolated spikes, the mixture shows a proliferation of virtual states starting at approximately – 5.5 eV. This orbital crowding effectively bridges the energy gap between the occupied nano-chain framework and the dye molecules. This transformation proves that the Hibiscus-derived N-CNCs do not just support the dye physically; they facilitate the rapid leap of electrons required for catalytic azo-bond cleavage. This dense population of states near the frontier region is the quantum driver behind the high-sensitivity pH signaling and the instantaneous catalytic response observed in the experimental phase. Furthermore, the E_T_ of the system becomes increasingly negative during the formation of the MO/N-CNCs complex (–1918.13 au), which is a classic indicator of thermodynamic stability. This confirms that the dye isn’t just floating near the nano-chains; it is forming a stable, energetically favorable partnership. The chemical softness (σ) surges from 0.2341 eV to 2.2668 eV, proving the system becomes highly reactive and ready for electron transfer. This explains the naked-eye sensitivity observed in the lab: the nano-chains act as an electronic reservoir, lowering the energy barriers of the dye and allowing the system to respond to pH changes and catalytic triggers with remarkable speed and visual clarity.

Based on the structural data, a plausible chemical mechanism for the morphological evolution of the interconnected 1D beads-on-a-string nano-chain architecture can be deduced through classic oriented attachment and dipole-driven self-assembly theories. During the high-energy microwave-assisted hydrothermal phase, NaOH serves as a powerful alkaline cutting agent, hydrolyzing the rigid, intra-molecular hydrogen-bonded lignocellulosic and pectin walls of the Hibiscus biomass into open-chain oligosaccharide fragments. Concurrently, the rapid thermal decomposition of urea introduces a dense concentration of active nitrogenous intermediates. These intermediates condense with the newly exposed carbohydrate fragments via covalent amide and amine linkages, as mathematically validated by the exceptionally high calculated DS = 2.75. This intensive, asymmetric surface functionalization induces an electronic imbalance across the primary carbonaceous clusters. To minimize the surface free energy of the highly polar aqueous colloidal environment, the individual ultra-small beads (2.08–2.96 nm) actively resist isotropic, random agglomeration. Instead, they undergo directionally guided linear alignment along their electrostatic dipole axes. This oriented attachment pathway fuses the independent clusters into stable, continuous 1D nano-chains, effectively creating the delocalized electronic highway required for rapid environmental monitoring and catalytic remediation. When paired with the highly economical, waste-to-wealth 'Kitchen-to-Lab’ synthesis from *Hibiscus* biomass, the platform establishes a highly practical, cost-effective, and chemically resilient liquid-phase treatment system that bypasses the need for energy-intensive catalyst recovery phases.

### Limitations and scope of analysis

To evaluate the operational performance of the synthesized Hibiscus-derived N-CNCs, a quantitative benchmarking analysis was performed against representative catalytic frameworks reported in recent literature (Table 4). This comparison tracks critical performance indicators, including the target dye matrix and degradation efficiency relative to time. Traditional heterogeneous systems, such as ternary GO/Gd/ZnO nanocomposites and bismuth-based metal–organic frameworks (i.e. BiPO_4_/NH_2_-MIL-53(Fe) and Bi5Ti_3_FeO_15_), achieve high degradation metrics but are characterized by longer operational timeframes, typically ranging from 30 to 240 min. These systems are often limited by mass-transfer resistance and the kinetics of interfacial charge separation in multi-phase boundaries. In contrast, carbonaceous materials such as reported carbon dots demonstrate faster degradation kinetics. Our synthesized Hibiscus-derived N-CNCs achieve a 79.30% chromophore degradation efficiency of MO.

**Table 4:** Quantitative comparison of dye degradation efficiencies across recent literature vs. this work.

Catalyst material	Dye type	Percentage (%)	Degradation time	Reference
GO/Gd/ZnO	Indigo carmine	96%	30 min	^44^
BiPO_4_/NH_2_-MIL-53(Fe)	Indigo carmine	94%	120 min	^45^
Bi5Ti_3_FeO_15_	Indigo carmine	97%	240 min	^46^
Carbon dots	Indigo carmine	91.2%	4.5 min	^47^
Carbon quantum dots surface-decorated TiO_2_ nanocomposites	MO	99%	150 min	^48^
Carbon dots from Hibiscus	MO	79.30	< 1 s (Instantly)	This study

It is important to note that the sub-second degradation observed in this study represents the resolution limit of our current experimental configuration. While these results suggest a highly efficient electron-injection mechanism enabled by the 1D interconnected morphology and nitrogen-doped nodes, this study does not claim complete mineralization into inorganic gases, as the analytical scope was limited to spectrophotometric degradation monitoring rather than TOC or gaseous-phase analysis. Furthermore, while the N-CNCs demonstrate rapid performance in a batch-mode, laboratory-scale setting, future studies are required to evaluate their efficacy against varied industrial effluent compositions and their long-term recyclability in continuous-flow systems. Despite these limitations, the N-CNC platform offers a promising metal-free, rapid-response alternative to conventional multi-phase catalysts, warranting further investigation into its scalability and long-term chemical stability.

## Conclusions

In summary, multi-functional nitrogen-doped carbon nano-chains (N-CNCs) were successfully engineered from repurposed *Hibiscus sabdariffa* botanical waste via a rapid, one-pot microwave-assisted hydrothermal route, presenting a sustainable, metal-free paradigm for advanced wastewater treatment. Morphological profiling via high-resolution TEM and SAED confirmed a definitive structural transition from isolated zero-dimensional architectures into a highly stable, interconnected one-dimensional (1D) beads-on-a-string carbon matrix. The individual carbonaceous beads exhibited extreme monodispersity with diameters strictly confined between 2.08–2.96 nm, anchored within a short-range turbostratic graphitic core rich in active disordered edge defectsThe catalyst drove a clean, permanent disruption of the MO chromophore framework almost instantly (< 1 s) upon contact, achieving peak spectrophotometric degradation efficiencies of 79.30% under acidic conditions (pH 3) and 74.67% under alkaline conditions (pH 12) via the irreversible reductive cleavage of the central –N = N– azo linkage. Quantitative surface profiling via FTIR spectroscopy verified a highly functionalized, hydrophilic matrix containing abundant amine, amide, and carbonyl networks. The integrated absorbance intensity ratio between the nitrogenous and aliphatic vibrational bands (A_C–N_/A_C–H_) yielded an elevated DS of 2.75. This exceptionally high DS value mathematically confirms a dense surface matrix of active chemical nodes, ensuring superb colloidal dispersibility and structural resilience while entirely eliminating the operational risk of toxic secondary metal leaching common to conventional transition-metal catalysts. Quantum–mechanical modeling via Density Functional Theory (DFT) and Density of States (DOS) analysis successfully decoded the electronic logic governing this dual-mode performance. Interfacial alignment and molecular coordination between the target dye and the nitrogenous nano-chain nodes trigger a massive collapse of the frontier molecular orbital energy gap within the hybrid matrix. This continuous electronic bridge enables near-instantaneous, non-radiative electron transport along the 1D carbon nano-chain highway. This sustainable, waste-derived liquid platform provides an elegant, highly competitive, and economically viable strategy for real-time monitoring and rapid purification within modern industrial water treatment infrastructures.

## Data Availability

All data is presented in the figures, graphics and tables in the context of the manuscript.
